# Why Age Matters: A Mixed-Methods Analysis of Intergenerational Contact With Older Adults With Dementia

**DOI:** 10.1093/geroni/igaa057.3053

**Published:** 2020-12-16

**Authors:** Keisha Carden, Sarah Letang, Jaimie Choi, Daniel Potts, Rebecca Allen

**Affiliations:** 1 University of Alabama, Tuscaloosa, Alabama, United States; 2 The University of Alabama, Tuscaloosa, Alabama, United States; 3 Tuscaloosa VA Medical Center, Tuscaloosa, Alabama, United States; 4 Alabama Research Institute on Aging, Tuscaloosa, Alabama, United States

## Abstract

This integrated mixed methods analysis examined outcomes for students enrolled in an experiential course (BATL), a didactic psychology of aging course, and introductory psychology courses. Students in the experiential course showed increased empathy, F(2, 345) = 29.058, p = .000 (M = 47.52, SD = .75), improved attitudes towards persons with dementia (PWDs), F(2,355) = 8.98, p < .0001 (M = 14.25, SD = .36), and greater increased interest in working with older adults, F(2,361) = 30.228, p=.000 compared to the other courses. A qualitative phase II follow-up explanations model (Creswell et. al., 2003) of participants’ journals using a hermeneutic coding process was employed to explore underlying mechanisms for such changes. These identified underlying mechanisms have significant implications for increasing interest and ability among students to work effectively with older adults with and without cognitive impairment. Further, this information yields insights for addressing workforce shortages in geriatric care.

